# Kenyan endemic bird species at home in novel ecosystem

**DOI:** 10.1002/ece3.2038

**Published:** 2016-03-14

**Authors:** Jan Christian Habel, Mike Teucher, Dennis Rödder, Marie‐Therese Bleicher, Claudia Dieckow, Anja Wiese, Christina Fischer

**Affiliations:** ^1^ Terrestrial Ecology Research GroupDepartment of Ecology and Ecosystem ManagementSchool of Life Sciences WeihenstephanTechnische Universität MünchenD‐85354FreisingGermany; ^2^ CartographyDepartment of Spatial and Environmental SciencesUniversität TrierD‐54286TrierGermany; ^3^ Zoologisches Forschungsmuseum Alexander KoenigD‐53113BonnGermany; ^4^ Restoration EcologyDepartment of Ecology and Ecosystem ManagementSchool of Life Sciences WeihenstephanTechnische Universität MünchenD‐85354FreisingGermany

**Keywords:** Agriculture, birds, effective habitat size, habitat connectivity, Kenya, land‐use, *Lantana camara*, novel ecosystem, potential habitat size, riparian vegetation, telemetry

## Abstract

Riparian thickets of East Africa harbor a large number of endemic animal and plant species, but also provide important ecosystem services for the human being settling along streams. This creates a conflicting situation between nature conservation and land‐use activities. Today, most of this former pristine vegetation is highly degraded and became replaced by the invasive exotic *Lantana camara* shrub species. In this study, we analyze the movement behavior and habitat use of a diverse range of riparian bird species and model the habitat availability of each of these species. We selected the following four riparian bird species: Bare‐eyed Thrush *Turdus tephronotus*, Rufous Chatterer *Turdoides rubiginosus*, Zanzibar Sombre Greenbul *Andropadus importunus insularis,* and the Kenyan endemic Hinde′s Babbler *Turdoides hindei*. We collected telemetric data of 14 individuals during a 2 months radio‐tracking campaign along the Nzeeu River in southeast Kenya. We found that (1) all four species had similar home‐range sizes, all geographically restricted and nearby the river; (2) all species mainly use dense thicket, in particular the invasive *L. camara*; (3) human settlements were avoided by the bird individuals observed; (4) the birds' movement, indicating foraging behavior, was comparatively slow within thickets, but significantly faster over open, agricultural areas; and (5) habitat suitability models underline the relevance of *L. camara* as suitable surrogate habitat for all understoreyed bird species, but also show that the clearance of thickets has led to a vanishing of large and interconnected thickets and thus might have negative effects on the population viability in the long run.

## Introduction

One of the main driver of global biodiversity loss is the destruction of pristine habitats and the subsequent fragmentation and disturbance of the remaining habitat patches (Sala et al. 2000; Watson et al. 2005; Smith et al. 2011). The fragmentation of previously interconnected and intact habitats causes the decrease in habitat size, and the increase in geographic isolation among remaining patches, as well as the deterioration of habitat quality due to negative edge effects (Fahrig 2003). In addition to these changes in the original habitat configuration (size, isolation, shape of remaining patches), disturbed habitat remnants may become modified by the invasion of exotic species which replace pristine vegetation – and subsequently create a novel ecosystem (cf. Lindenmayer et al. 2008; Hobbs et al. 2013). Among these environmentally driven extrinsic factors, intrinsic drivers such as habitat demands (e.g., need of specific resources, microhabitat structures, species interactions) and species' behavior (e.g., sedentary dispersal) affect species' persistence – in particular in fragmented and modified habitats (Devictor et al. 2008). Both, extrinsic and intrinsic factors have to be taken into consideration when conducting effective conservation management, especially in regions dominated by the human being, and novel ecosystems (Andrén 1994; Sutherland et al. 2004).

Loss of sensitive, pristine ecosystems, and its transformation into novel ecosystems dominated by invasive exotic vegetation can frequently be observed in areas where human demographic pressure is high, and abiotic conditions are favorable for agriculture (Smith et al. 2011). The riparian thickets along East African rivers represent such a fragile ecosystem, providing important habitat retreats for many (endemic and endangered) plant and animal species. In parallel, these riparian ecosystems also provide manifold ecosystem services (fertile soil, high standing ground water, wood and shadow) (McClanahan and Young 1996). The increasing human population in combination with mal‐adapted land‐use techniques causes conflicts between wildlife conservation and the depletion of natural resources by the local human population (Enanga et al. 2011). Subsequently, riparian thickets occur today highly fragmented, and the small and isolated remnants are disturbed and often invaded or even completely replaced by the exotic shrub species *Lantana camara*, which may provide an important surrogate habitat for many rare and endangered riparian species.

Radio‐tracking data may provide important information about the use of space by animals, potential territories and home‐range sizes, and species' specific movement behavior. Such data allow estimating species' habitat use and home‐range sizes which might be of relevance for its long‐term persistence. Furthermore, data from movement ecology give insight into stabilizing and equalizing mechanisms shaping biodiversity, for example, interactions among individuals, species, and communities (Jeltsch et al. 2013). And finally, such data can be used to train models to project the distribution of suitable habitats for observed species (Denoel and Ficetola 2015). Such real‐world, small‐scale observations in combination with large‐scale projections are of relevance in particular in landscapes being characterized from rapid habitat destruction and disturbance, and its large‐scale replacements by novel ecosystems. Furthermore, such data may create the background to improve nature conservation by creating corridors and stepping stones at selected sites.

In this study, we conducted radio‐tracking to analyze the habitat use and movement behavior of four East African riparian bird species, the Bare‐eyed Thrush *Turdus tephronotus*, Rufous Chatterer *Turdoides rubiginosus*, Zanzibar Sombre Greenbul *Andropadus importunus insularis*, and the Kenyan endemic and vulnerable Hinde′s Babbler *Turdoides hindei* (cf. Zimmerman et al. 1996; BirdLife International 2015). Our study framework incorporates in total three rivers which are characterized by different degrees of habitat quality, with two rivers providing still intact pristine thickets, while the vegetation along the third river is strongly invaded by the exotic *L. camara* shrub. Based on our telemetry data and a detailed land‐cover map (collected at one of these three rivers), we run models on species' habitat suitability for each species and for the three rivers. In detail we ask the following questions:

1. Do habitat use and home‐range sizes differ significantly among the four species analyzed?
2. Do habitat structures affect the movement behavior of the four bird species?
3. Does habitat quality (configuration and type of thicket) have any effect on the occurrence of the bird species observed?
4. Which conservation strategies can be drawn from our results?

## Methods

### Study region

Our study region is located in a semiarid part of south‐eastern Kenya, close to Kitui city (1.421017°S, 38.024145°E). The study region is located at about 1050 m a.s.l. and characterized by about 1079 mm average annual rainfall. Annual uncertain rainfall conditions are divided into two short to very short discrete rainy seasons with a two‐third reliability of 250–300 mm and 400–450 mm respectively. These rainy seasons are dictating the agricultural practices with growing periods ranging from 75 to 85 days and 85 to 105 days, respectively (Ministry of Agriculture, 2006). Radio tracking was conducted along the Nzeeu River (tributary of Tana River). Models of habitat suitability were projected for Nzeeu River and the two neighboring rivers, Kalundu and Ithiani. These three rivers show different degrees of habitat disturbance: The Nzeeu River provides a fine‐grained mosaic of agricultural plots and thickets, dominated by the exotic *L. camara*, which invaded and replaced the pristine vegetation (but also the other rivers to a lower extent). Rapid invasion of this plant species can be observed over major parts of East Africa along rivers, since the 1950s (Lyons 2000). The two other rivers, Kalundu and Ithiani, still harbor large and intact indigenous riparian thickets. Many people are settling along these rivers to conduct subsistence agriculture (Ministry of Agriculture, 2006). The fast growing human population (2.4% population growth rate throughout the past 20 years, Kenya National Bureau of Statistics, 1999 and 2009) caused a rapid and ongoing transformation of dense riparian thickets into open agricultural land.

### Study species

The four focal bird species are mainly found in thickets over major parts of East Africa (Bennun et al. 1996; BirdLife International 2015). The Bare‐eyed Thrush *T. tephronotus* is found in dry woodlands and thickets from the coastal shrubs to 1600 m a.s.l. (Zimmerman et al. 1996). The species feeds on insects, fruits, and seeds, frequently foraging on the ground inside of dense vegetation (Del Hoyo et al. 2014). The Rufous Chatterer *T. rubiginosus*, mostly common in dry coastal lowlands below 1500 m a.s.l., is foraging on the ground or in low shrubbery, feeding on invertebrates as well as mango and papaya rinds (Zimmerman et al. 1996). This species appears in Kenya in two subspecies (Zimmerman et al. 1996), with *T. r. rubiginosus* found in our study region (Del Hoyo et al. 2014). The Zanzibar Sombre Greenbul *Andropadus importunus insularis* is exclusively found along the coastal lowlands of southeastern Kenya and along the rivers toward the inland (Zimmerman et al. 1996; Del Hoyo et al. 2014). The vulnerable Kenyan endemic Hinde′s Babbler *T. hindei* is only found in dense riparian thickets along few rivers in the semiarid, southeastern Kenyan lowlands (but also at higher elevations) (Shaw et al. 2003, 2013). Agricultural intensification has caused a destruction of habitats and subsequently a severe decline of this insectivorous bird species (Shaw et al. 2013). The four target species differ in terms of diet (*Turdoides* are insectivorous, *Turdus* omnivorous, *Andropadus* frugivorous) and sociality (*Turdus* and *Andropadus* live in territorial pairs, *Turdoides* are cooperative breeders and live in flocks of 5–10 individuals) (Zimmerman et al. 1996; Habel et al. 2015).

### Radio tracking

Radio tracking was conducted during two periods, 1 month in August 2014 and 1 month in March 2015. Bird individuals were caught with mist nets along the Nzeeu River. Individual measurements (body mass, size of head, tarsus, and wing) were taken. The birds were individually ringed and equipped with a Pip Ag376 backpack tag, sending 25 ms signals at a pulse rate of 50 ppm, with a predicted life span of 32 days (Biotrack Ltd, Wareham, Dorset, UK). Weight of the tags was about 1.5 g and thus below the accepted threshold value of 4–5% of the body weight for all study species (Kenward 2001). Tags were attached at the birds with wing‐loop harnesses made of rubber band and fixed with superglue. Signals were received with a four‐element Yagi‐antenna (HB9CV) (Wagener Telemetrieanlagen, Cologne, Germany) and a tracking receiver R1000 with a 148–174 MHz band width (Communication Specialists Inc., Orange, Canada). Birds were tracked by triangulating the tag's position. In order to do so, two observers took a bearing simultaneously every 10 min from 7:00 am to 5:30 pm using a compass and recorded their own position with a GPS device. All observed individuals were first traced the day after catching, so that the individuals were able to adapt to the transmitter and to prevent short‐term behavioral changes (Kenward 2001). As both study periods cover the end of a dry season the two data sets were merged.

### Land‐use mapping

For detailed land‐cover mapping, an Acer Iconia tablet‐PC coupled with an external Bluetooth GPS data logger device and the free GIS software Quantum GIS 2.4.0 (QGIS) was used (QGIS Development Team, 2014). The following land‐use characteristics were assessed as lines, points, or polygons: line geometry – river, roads and paths; point data – settlements; polygon geometry – indigenous thicket, mixed thicket (indigenous with *L. camara*), pure *L. camara* thicket, low growing crops up to growing height of about 80 cm (in the following “crops low”, e.g., tomatoes, kales, cowpeas, beans) and high‐growing crops taller than 80 cm (in the following “crops high”, e.g., maize, pigeon peas, fodder crops), and settlements. These data were postprocessed using QGIS functions to validate geometries, to complete geometric topology of polygons and lines, and to standardize assignment of objects according to our mapping criteria. This detailed land‐use map was developed for the area where we conducted our telemetry study. For the three rivers for which we calculated habitat suitability models, we digitized the following habitat types along a 400 m buffer around the three rivers: river, roads, settlements, thickets, open/agricultural land.

### Home‐range sizes, habitat preferences, and movement behavior

Positions of animals were calculated from both bearings using the Microsoft Excel (2010) spreadsheet according to Hodgson (2013). Positions located more than 1000 m distant from the observer positions were not taken into consideration for further analyses due to potential tracking errors. Prior to home‐range analysis, we plotted home‐range area per individual against sample size (days after start of data acquisition) (Gese et al. 1990), as sample size may affect home‐range size estimates (e.g., Harris et al. 1990). As the estimated area did not increase as more locations were added, we assume that sample sizes suffice to reliably predict birds' home‐range sizes (shown in Fig. S1).

Individual home‐range sizes were calculated using the minimum convex polygon (MCP) estimator using 95% of the relocations which were closest to the centroid of the home range using the R package adehabitatHR (Calenge 2006). The MCP method was selected as this estimator is comparable to other estimators (Kernohan et al. 2001). Further, Kernel home ranges for 95%, 75%, and 50% levels were calculated for the estimation of the utilization distribution using the ad hoc method for the calculation of the smoothing parameter href (Worton 1995). The Kernel method was used as it requires a low amount of data points to calculate stable home ranges, is robust with respect to autocorrelations, and multiple activity centers can be calculated (Kernohan et al. 2001). For further analysis and visualization, home‐range contours were exported to QGIS using the R package maptools (Bivand et al. 2014).

To test for potential habitat preferences, the 75% Kernels (K75) within the birds' core area were intersected with the detailed land‐cover map. Percentages of each habitat type within the birds' home ranges were calculated (r: proportion of habitat used). Further, percentages of each habitat type within the whole study area were calculated (p: proportion of habitat available). To identify preferred and avoided habitat structures, the Jacobs Index (*D*) based on the proportions used and available was calculated by the following formula: *D* = (*r* − *p*)/(*r* + *p* − 2*rp*). This index ranges from −1 (total avoidance) over 0 (no interaction) to 1 (absolute preference) (Jacobs 1974). To test for significant preference or avoidance of the different habitat structures, Jacobs' indices per habitat structure were tested using the sign test because of non‐normal error distribution (Zar 1996) against a median of 0 (no interaction) implemented in the R package BSDA.

Movement distances between subsequent locations of individuals (in m·10 min^−1^) were calculated using the R package adehabitatLT (Calenge 2006). To analyze movement behavior in relation to the different habitat types, individual movement distances were calculated within the following habitats (with *n *>* *50): mixed thicket, *L. camara*, crops low and crops high, using the exact habitat type of bird location determined by the QGIS “point‐in‐polygon function.”

### Statistics

First, to test for differences in home‐range sizes among species (*T. tephronotus* vs. *T. rubiginosus* vs. *A. importunus insularis* vs. *T. hindei*), we performed a one‐way ANOVA for each home‐range size estimate separately. To fit model requirements, home‐range sizes were tested for normal error distribution prior to the analysis using a Shapiro–Wilk normality test. In case of non‐normality of errors, home‐range size was log(*x* + 1)‐transformed.

Second, differences between habitat structures and species, as well as two‐way interactions, were tested using linear mixed‐effects models (Pinheiro and Bates 2000) with a maximized log‐likelihood implemented in the nlme R package (Pinheiro et al. 2012). Preferences or avoidance (Jacobs index) and movement distances were used as response variables. To account for differences between bird individuals, as well as for repeated measurements of movement distances of the same individual, the factor birdID (*n* = 14) was included as a random effect. Different variances per habitat structure, species, or ecological groups were modeled using the varIdent variance structure. Models with different within group variances were compared by choosing the lowest AIC (Akaike information criterion) value from an ANOVA table (Pinheiro and Bates 2000). To achieve a normal error distribution and/or to avoid heteroskedasticity, movement distances were log(*x* + 1)‐transformed. Finally, model simplification was carried out in a backward stepwise model selection procedure by AIC implemented in the R package MASS (Venables and Ripley 2002) until minimal adequate model was obtained using the “stepAIC” function. Significance of terms in the best model was assessed by calculating the *F*‐ and *P*‐values of an ANOVA table. Contrasts between species as well as habitat structures were investigated by re‐ordering factor levels. For all analysis we used R version 3.0.2 (R Core Team 2013). In the text and figures, nontransformed means and standard errors are presented.

### Null model home ranges

To test whether home ranges reflect site fidelity rather than nomadic movement, we compared the actual home‐range size in terms of K75 kernel density estimates and MCP95s with null distributions obtained from “random walk models” (RWMs): For each specimen, we computed 1000 simulations based on a bootstrap approach using randomizations of the respective turning angle and distances between successive fixes of the real radio‐tracking data (see also Munger 1984; Spencer et al. 1990; Ghaffari et al. 2014). All calculations were performed in R using the following packages: adehabitatHR, adehabitatLT (Calenge 2006), SDMTools (Van Der Wal et al. 2014), maptools (Bivand and Lewin‐Koh 2015), raster (Hijmans 2015), and fields (Nychka et al. 2015). Site fidelity was assumed when the observed home‐range sizes were lower than the 95% confidence intervals of the RWMs (Munger 1984; Spencer et al. 1990).

### Habitat suitability models

To project the spatial distribution of suitable habitats for each of the four species along the three rivers, we used RapidEye aerial satellite imagery (Blackbridge 2014). All imagery was acquired on 13th August 2014 with a total coverage of 625 m² resulting in a spatial resolution of 5 m pixel size with a spatial accuracy of 10 m (Blackbridge 2014). Imagery was delivered as orthorectified raster images including five remote sensing channels (Blue: 440–510 nm, Green: 520–590 nm, Red: 630–685 nm, Red Edge: 690–730 nm, NIR: 760–850 nm wave lengths) and a cloud cover percentage of 6%.

Based on the five raw remote sensing channels (Blue, Green, Red, Red Edge, and NIR), we converted the radiance values to top of the atmosphere reflectance (TOA) according to the product specifications. This procedure removes the effects of illumination, orientation, and position of the target and allows a quantification of the reflectance of the observed objects, which is generally more suitable than radiance images to compute vegetation indices (Jackson and Huete 1991). As suggested by Ortiz et al. (2013), the following seven indices were computed: NDVI [(NIR − Red)/(NIR + Red)], Red Edge Green NDVI [(Red Edge −  Green)/(Red Edge + Green)], GNDVI [(NIR − Green)/(NIR + Green)], NDRE [(NIR − Red Edge)/(NIR + Red Edge)], Chlorophyll Green Model (GCM) [(NIR/Green) − 1], Chlorophyll Red Edge Model (GRM) [(NIR/Red Edge) − 1], and Red Edge NDVI [(Red Edge − Red)/(Red Edge + Red)]. All computations were performed using the raster and landsat packages (Goslee 2011, Hijmans 2015) in R.

Based on our detailed land‐cover map, we computed the minimum distance of mixed thicket vegetation and settlement from the river for each grid cell of the study area. These three variables were used in the modeling framework to quantify the spatial configuration of the preferred habitats.

For SDM development, we used MAXENT 3.3.3k (Phillips et al. 2006) allowing linear, quadratic, and product features. For model calculations, we pooled individuals from each taxon into a species group (in total four species groups). For each species group we computed 10 models randomly splitting the pooled radio‐tracking records in 70% which were used for model training and 30% used for model evaluation using the “area under the receiver operating characteristic curve” (AUC). For further processing, the average predictions of the 10 models were used.

As previous trials revealed a strong dominance of distance to river affecting the probability of occurrence (variable importance > 80%), we decided to restrict the training range of the models within the preferred distance to rivers in order to capture fine‐scale habitat features. Therefore, in a first step, we restricted the environmental background to an area with a MCP enclosing the pooled radio‐tracking data and used distance to river as single predictor. The resulting models were reclassified to presence/absence maps applying a 10 percentile training omission threshold and subsequently used as mask files in the modeling procedures to capture the spatial dependencies of each species to river ecosystems.

**Figure 1:**
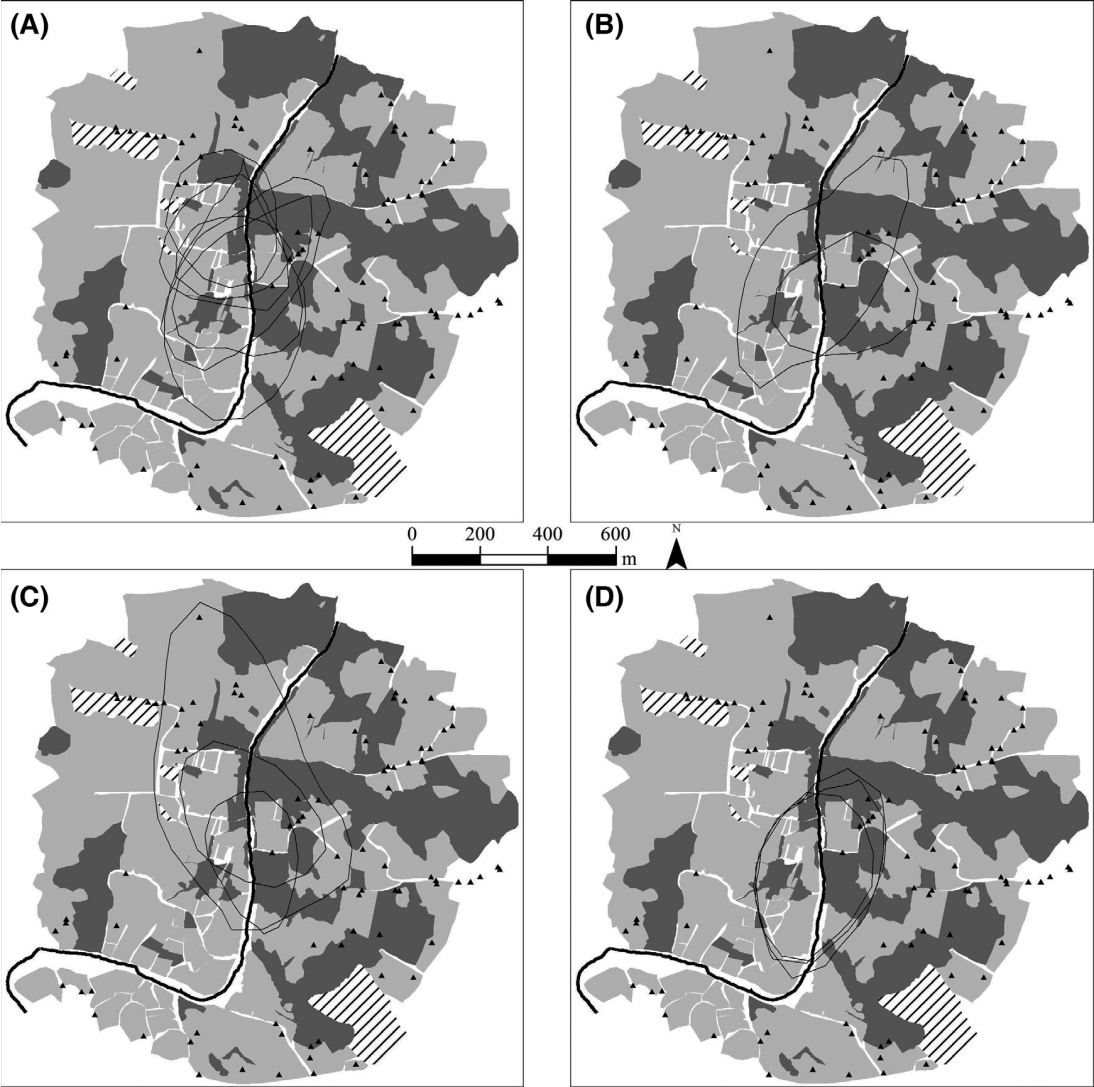
Distribution of the 75% Kernels shown for each individual by black lines, analyzed for the four study species: (A) *Turdus tephronotus*, (B) *Turdoides rubiginosus,* (C) *Andropadus importunus insularis*, and (D) *Turdoides hindei*. Background map indicates the land‐cover structures, with thicket (dark gray), agricultural land (light gray), settlements (black–white shaded and black triangles). The Nzeeu River is shown as bold black line.

A detailed land‐use map quantifying *L. camara* cover was only available for a smaller subset of the study area (see Fig. 1). Hence, we used the same modeling approach as described above to develop a detailed prediction of this vegetation type within the general study area: We used randomly generated records within the *L. camara* spots shown in Figure 1 and the vegetation indices derived from RapidEye as environmental predictors. The logistic probabilities obtained from MAXENT were used as environmental predictor in the final models.

For each of the four species, we trained MAXENT models within the intersection of the MCPs obtained from the telemetry records and the reclassified MAXENT model taking distance to river into account. As environmental predictors, we included all vegetation indices, the prediction of *L. camara* as well as distance to mixed thicket vegetation and settlements.

## Results

### Land‐cover assessment

In total, we assessed 148.2 ha of land, consisting of the following categories: 97.2 ha (65.6%) agricultural land (with 13.9 ha, i.e., 9.4% “crops low”; 83.3 ha, i.e., 56.2% “crops high” and 44.6 ha, i.e., 30.1% thicket). The thicket cover was dominated by the exotic *L. camara* with 41.9 ha, (94.2%) of the total thicket cover, and 9.6 ha, (21.6%) being mixed (*L. camara* with indigenous thicket). Only 2.6 ha, that is, 5.9% can be classified as pure indigenous thicket. Human settlements cover 6.4 ha, that is, 4.3% of the total area assessed.

The land‐cover assessment along the three rivers based on RapidEye aerial satellite imagery (the background for our habitat suitability model) showed the following landscape composition for a 400 m buffer along each side of the river: Nzeeu River with 13.6% thicket, 85.6% open land, 0.8% settlement; Kalundu River with 8.8% thicket, 90.5% open land, 0.7% settlement; Ithiani River with 10.1% thicket, 89.9% open land and 0.02% settlement.

### Home ranges

Radio tracking was conducted for a total period of 37 days, with 399 ± 55 fixes and 10 ± 1 days of observations collected per individual, and 414 ± 90 fixes for each species (*T. tephronotus*:* n* = 6; *T. rubiginosus*:* n* = 2; *A. importunus insularis*:* n* = 3; *T. hindei*:* n* = 3; Table 1). Mean home‐range sizes were 52.8 ± 5.8 ha for the MCP95 estimator, 61.8 ± 4.4 ha for K95, 14.9 ± 1.9 ha for K75, and 5.8 ± 0.8 ha for K50 (for a detailed list of home‐range sizes per individual and species, see Table 1). Home‐range sizes for the different home‐range estimators did not differ among the four species (Table 1).

**Table 1:** Home‐range sizes (in ha) calculated by the different estimators for individuals of the four study species (mean values with standard errors). The table shows the respective species name, abbreviation of individuals observed, duration of observation, number of fixes (*N*), the 95% minimum convex polygon (MCP95), the smoothing parameters for the Kernel home‐range estimates, and the 95% Kernel (K95), 75% Kernel (K75), and 50% Kernel (K50) home‐range sizes. Differences between species were calculated using one‐way ANOVA. Degrees of freedom, *F*‐ and *P*‐values are given

Species Individual	*T. tephronotus*						*T. rubiginosus*		*A. importunus insularis*			*T. hindei*			Differences between species		
	BT1	BT2	BT3	BT4	BT5	BT6	RC1	RC2	ZG1	ZG2	ZG3	HB1	HB2	HB3	df	*F*	*P*
Duration of observation	2014‐08‐08 –14	2014‐08‐11 –18	2014‐08‐16 –22	2014‐08‐19 –24	2015‐02‐27 –03‐15	2015‐03‐04 –20	2015‐02‐28 –03‐17	2015‐03‐11 –20	2014‐08‐10 –17	2014‐08‐16 –21	2014‐08‐19 –24	2015‐03‐02 –20	2015‐03‐07 –20	2015‐03‐10 –20			
*N*	298	225	211	202	628	561	527	346	289	211	147	819	623	501			
Home‐range estimator																	
MCP95	37.87	33.90	35.05	18.07	54.94	75.88	57.74	63.02	50.58	16.39	58.57	92.57	68.39	75.60			
	42.62 ± 7.48						60.38 ± 1.87		41.85 ± 10.56			78.86 ± 5.85			3, 10	3.12	0.07
href	55.79	61.88	84.06	50.83	55.67	66.12	56.93	76.08	64.50	52.79	100.59	62.11	65.25	67.99			
K95	52.05	48.62	70.06	29.45	53.53	71.55	65.26	71.79	61.38	31.29	89.64	77.58	70.68	72.72			
	54.21 ± 5.78						68.52 ± 2.31		60.77 ± 13.76			73.66 ± 1.67			3, 10	0.97	0.44
K75	10.63	10.08	21.09	6.50	12.56	16.16	11.35	19.33	12.92	8.28	37.01	15.32	15.81	12.77			
	12.84 ± 1.91						15.34 ± 2.82		19.40 ± 7.27			14.64 ± 0.77			3, 10	0.29	0.83
K50	3.92	4.74	7.53	2.60	5.29	6.15	3.78	7.59	4.85	3.22	15.63	5.75	5.58	3.93			
	5.04 ± 0.64						5.69 ± 1.35		7.90 ± 3.18			5.09 ± 0.47			3, 10	0.24	0.86

### Habitat preferences

The K75 home ranges calculated for the 14 individuals of the four species show a strong restriction to the river bed and the riparian vegetation (Fig. 1). The Jacobs Index shows significant differences among preferences or avoidance of the different habitat types (*F*_1/5_ = 312.02, *P* < 0.001, with *L. camara* > crops low > crops high > mixed thicket > settlements = indigenous). Thereby, the exotic *L. camara* (0.75 ± 0.02, *s* = 14, *P* < 0.001), as well as “crops low” (0.32 ± 0.04, *s* = 14, *P* < 0.001), were preferred, while “crops high” (−0.31 ± 0.03, *s* = 0, *P* < 0.001), mixed thickets (−0.57 ± 0.06*x*,* s* = 0, *P* < 0.001), settlements (−0.91 ± 0.05, *s* = 0, *P* < 0.001), as well as indigenous vegetation (−1.00 ± 0.00, *s* = 0, *P* < 0.001), were avoided (remark: the latter two parameters were not available in direct adjacency of the river bed). We found no significant differences concerning habitat preferences (interaction terms, as well as species excluded from the minimal adequate models).

### Movement behavior

**Figure 2:**
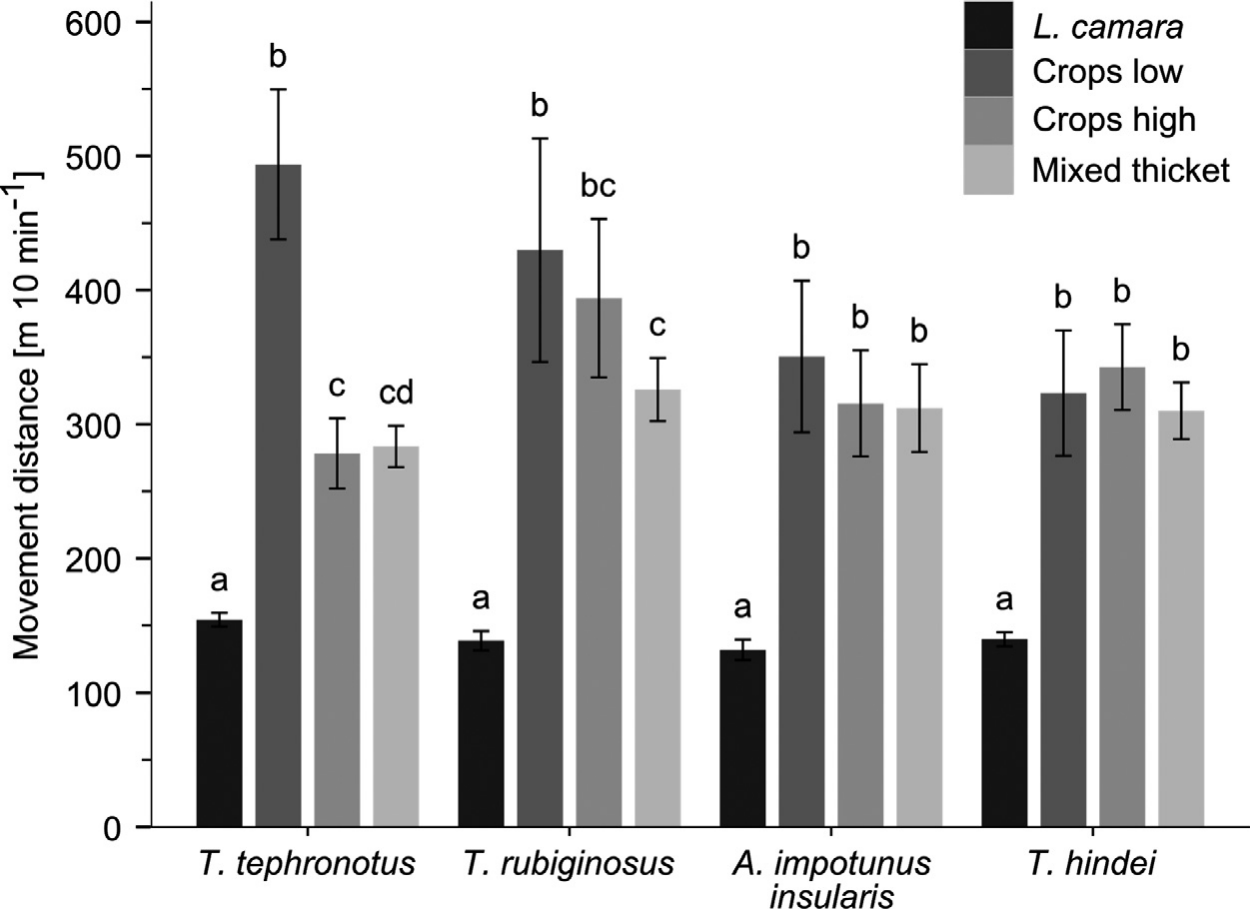
Movement distances in m·10 min^−1^ in different habitat structures for the four study species *Turdus tephronotus*,* Turdoides rubiginosus, Andropadus importunus insularis*, and *Turdoides hindei*. Differences between habitat types per species are indicated by different letters (linear mixed‐effects models). For better visualization, nontransformed data are presented.

Movement distance differed significantly depending the type of habitat (*F*_3/4616_ = 243.29, *P* < 0.001). Birds showed shorter movement distances in *L. camara* (143.9 ± 3.0 m·10 min^−1^) compared with “crops low” (402.8 ± 30.4 m·10 min^−1^) and “crops high” (315.1 ± 17.7 m·10 min^−1^), or “mixed thicket” (299.8 ± 10.5 m 10 min^−1^). Further, movement distance varied with habitat structure and bird species (interaction habitat structure × species; *F*_9/4616_ = 3.45, *P* < 0.001). *T. tephronotus* showed significantly lower movement distances in mixed thicket = crops high < crops low, while for *T. rubiginosus,* we just found lower movement distances in mixed thicket compared to crops low. For *A. importunus insularis* and *T. hindei* movement distances were shortest in *L. camara* compared to all other habitat types (Fig. 2). However, there was no significant difference among the movement distances of the four species (*F*_3,10_ = 0.22, *P* = 0.88). We measured the following movement distances for the single species: *T. tephronotus*, 202.5 ± 5.7 m·10 min^−1^; *T. rubiginosus*, 194.9 ± 8.9 m·10 min^−1^; *A. importunus insularis*, 179.5 ± 9.3 m·10 min^−1^; and for *T. hindei* 189.1 ± 6.1 m·10 min^−1^.

### Habitat suitability model

**Figure 3:**
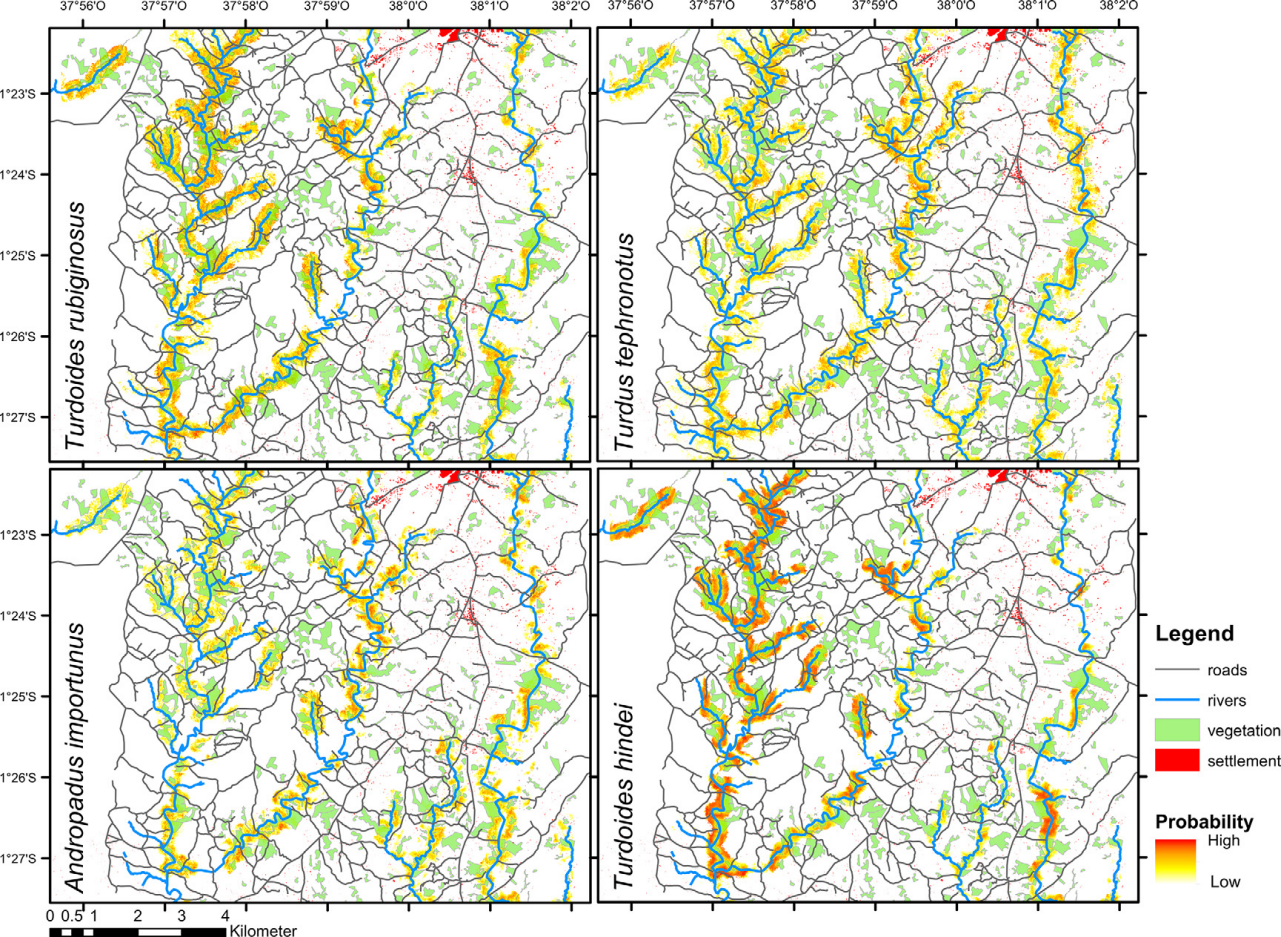
Habitat suitability models for the four study species, *Turdus tephronotus*,* Turdoides rubiginosus, Andropadus importunus insularis*, and *Turdoides hindei*. Warm colors predict areas of high habitat suitability.

Model performance was well above 0.7 in terms of test and training AUC in all models (Table 2). In the model developed for *L. camara*, the red band had the highest variable contribution (26.9%), followed by the green band (23.8%). All variable contributions are given in Table 2. In the models developed for the bird species, the variables with the highest contributions were “distance from settlements” and “distance from vegetation,” followed by probability of *L. camara* in *T. rubiginosus* and the *T. tephronotus*, but not in the other two species, where “distance from vegetation” was more important (Table 2). Further, the models predict a patchy distribution of suitable habitats – in particular for *T. hindei* (Fig. 3).

**Table 2:** Summary statistics of the distribution model developed for the distribution of *L. camara* and the habitat suitability models for the four bird species *Turdus tephronotus*,* Turdoides rubiginosus, Andropadus importunus insularis*, and *Turdoides hindei*

	*L. camara*	Final model			
		*T. rubiginosus*	*A. importunus* l	*T. hindei*	*T. tephronotus*
Training AUC	0.78	0.71	0.79	0.77	0.73
Test AUC	0.77	0.71	0.77	0.77	0.71
10 percentile training presence logistic threshold	0.31	0.29	0.21	0.20	0.26
Variable contribution (%)					
*L. camara*	–	18.9	4.0	2.1	12.2
Chlorophyll Green Model	1.9	0.3	0.7	0.7	1.6
Chlorophyll Rededge Model	0.5	1.4	1.1	0.5	1.3
GNDVI	0.1	0.5	5.4	1.5	1.6
Green	23.8	–	–	–	–
NDVI	0.3	1.8	0.5	1.6	0.5
NIR	15.1	–	–	–	–
Red	26.9	–	–	–	–
Red Edge	14.8	–	–	–	–
Red Edge Index	0.0	2.3	1.5	0.9	1.5
REGNDVI	6.1	7.6	8.5	1.5	9.4
RENDVI	10.6	3.0	3.0	0.6	2.7
Settlement distance	–	44.4	36.7	54.5	14.4
Vegetation distance	–	19.7	38.5	36.0	54.9

## Discussion

Our data indicate two factors being of key‐relevance for the occurrence of the four study species: close geographic proximity to the river, and the occurrence of dense thickets (underlined by the Jacobs Index and restricted movements within thickets). Hereby, the exotic invasive *L. camara* plays a central role, as this plant species provides a suitable surrogate habitat for most bird species studied here. In contrast, open land (here mainly agricultural plots) is avoided and birds cross this matrix by rather fast movements if compared with how they forage inside of *L. camara* thickets, where they mainly feed and rest (JCH own observations). Strong habitat patchiness becomes revealed by our habitat suitability model, indicating that thickets close to the river are important habitats (and corridors), while open land is rather unsuitable for these riparian bird species.

Our estimates of home‐range sizes are in line with the home‐range sizes observed for other East African forest bird species (Aben et al. 2012). Newmark et al. (2010), for example, found aggregated home ranges of more than 10 ha for various tropical mountain forest birds in the Usambara Mountains, Tanzania. The estimated home ranges in our study are spatially restricted to dense thickets along the river. This spatial restriction becomes further underlined by the Jacobs Index and the movements observed, with significantly longer time remaining inside than outside of thickets. The exotic invasive *L. camara* here acts as an important novel ecosystem which replaced the pristine riparian vegetation almost entirely in our study region since some decades (cf. Teucher et al. 2015) (discussed in detail below).

Several radio‐tracking studies showed strong impact of habitat destruction on local populations, with increasing home‐range sizes in environments of strong habitat fragmentation (Carey et al. 1990; Hansbauer et al. 2008). This coherence between habitat configuration and home‐range sizes was also observed for one of our targeted species, the Hinde′s Babbler in previous studies. Increasing thicket coverage had a positive effect on species' abundance and population density and caused a reduction in home‐range sizes (Shaw et al. 2003; Habel et al. 2015). Subsequently, these results show that home‐range sizes increases and family group size decreases due to habitat deterioration (Shaw et al. 2003). Thus, large proportions of open land may provide less food resources and thus may lead to increasing predation pressure and decrease in species abundance.

Apart from habitat configuration (habitat size, isolation, and the shape of patches), habitat quality may further affect the persistence of local populations (cf. Dennis and Eales 1997). All four studied bird species are mainly observed in the exotic invasive *L. camara*. These thickets provide food and protection against natural predators and the human being. After its invasion about 50 years ago (Lyons 2000), this plant species replaced most of the previous indigenous vegetation, after this became disturbed or completely cleared (Day et al. 2003). Today, this invasive thicket acts as important surrogate habitat for many endangered vertebrate species (Njoroge et al. 1998). High habitat quality in combination with comparatively specific ecological demands (here the need of dense thicket which provides protection from predators and resources for feeding and breeding) often cause restricted movements and a strong site fidelity, as shown for other East African bird species, like the Kulal White‐eye (Borghesio and Laiolo 2004) and four other East African cloud forest bird species (Habel et al. 2015). This coherence was also shown for other vertebrate and invertebrate species with specific habitat demands (Schneider 2003; Moore et al. 2008; Smith et al. 2011). Movement is energy consuming and thus only a positive investment if the new habitat patch provides an improved habitat (and thus life) quality (cf. Robles and Ciudad 2012).

The movement distances observed and calculated for different habitats strongly depend on the respective land cover and the time of observations, but might also depend on seasonal stages, and the phenology of species. Studies showed that observed home‐range sizes are smaller during periods with high resource availability (Wiktander et al. 2001). In our study, we combined and analyzed time cohorts collected during the end of two dry seasons (in March and August), which do not cover the breeding season of any of the four birds (Zimmerman et al. 1996). Observations during the rainy (and breeding) season might provide another picture of movements and home‐range sizes (see Jeltsch et al. 2013).

Home‐range sizes and movement behavior may further be affected from social behavior, like territories. Data obtained from our Kernel analyses showed inter‐ and intraspecific overlaps, and thus, bird individuals observed in our study are assumed to have no distinct territories, or being member of the same local family, except species of *Turdus* and *Andropadus*, which are living in territorial pairs, as well as species of the genus *Turdoides* which are cooperative breeders living in family groups (Teucher et al. 2015). Teucher et al. (2015) performed daily observations of single individuals of *T. hindei* over several weeks; home‐range sizes calculated based on these observation points showed much smaller home‐ranges than found in this telemetry study (7.42 ha from observation vs. 14.64 ha from telemetry). However, these diverging results might be due to observation bias, as observers are tending to search for a target species where it is most likely to find, while telemetry allows a rather nonbiased, more objective observation (which may lead to larger home‐range sizes). On the other side, direct observations may provide more precise locality and behavior data, which is of particular importance in such fine‐grained landscape mosaic, where triangulated occurrence points of birds may fail to correctly display the real position of occurrence.

The situation of fragmented riparian thicket gets revealed by our habitat suitability models, wherein the suitable habitat patches of the four species are all, to varying degrees, fragmented. The models correctly identified *L. camara* as main habitat for all four bird species. However, most of the remaining thicket patches are small and geographically isolated and thus might be not suitable for long‐term persistence for the observed species. Ongoing clearance of thickets may lead to increasing patch partitioning. Subsequently “potential habitat size” may strongly diverge from “effective habitat size” – as most of the remaining thicket patches are too small and too patchy and thus being not of any suitability for long‐term persistence of the observed bird species.

Practical conservation strategies for riparian thickets have to combine two fields of interest: the preservation of thickets along rivers for wildlife, but also the accessibility of land close to the river for the local human population, to cultivate food crops. Thus, “human–wildlife corridors” may combine human interests (agriculture, usage of resources) and wildlife concerns (but also corridors for invasive plant species like *L. camara*) along narrow strips of some tens of meters along rivers. In practice, such “human–wildlife corridors” should combine food crop production (e.g., pigeon peas, millets, other grains) with primary (or secondary – *L. camara*) habitat patches for wildlife. Such corridors may reconnect small and isolated thickets and thus contribute to an increase in the availability of the “effective habitat size”. This scenario might be the most realistic future to maintain both, intact ecosystem services for the human being as well as thickets to guarantee long‐term persistence of endangered species, at home in novel ecosystems.

## Conflict of Interest

None declared.

## Supporting information


**Figure S1**. Home range area (in ha) per individual against sample size (days after start of data acquisition), shown for (a) Minimum Convex Polygon estimator using 95% of the relocations (MCP95) in August 2014; (b) Kernel home ranges for 95% levels (K95) in August 2014, (c) MCP95 in February/ March 2015, and (d) K95 in February/ March 2015.

## References

[ece32038-bib-0001] Aben, J. , F. Adriaensen , K. W. Thijs , P. Pellikka , M. Siljander , L. Lens , et al. 2012 Effects of matrix composition and configuration on forest bird movements in a fragmented Afromontane biodiversity hot spot. Anim. Conserv. 15:658–668.

[ece32038-bib-0002] Andrén, H. 1994 Effects of habitat fragmentation on birds and mammals in landscapes with different proportions of suitable habitat: a review. Oikos 71:355–366.

[ece32038-bib-0003] Bennun, L. , D. Pomeroy , and C. Dranzoa . 1996 The forest birds of Kenya and Uganda. J. East Afr. Nat. Hist. 85:23–48.

[ece32038-bib-0004] BirdLife International . 2015 Available at: http://www.birdlife.org (accessed 14 April 2015).

[ece32038-bib-0005] Bivand, R. , and N. Lewin‐Koh . 2015 maptools: Tools for Reading and Handling Spatial Objects. R package version 0.8‐36. Available at: http://CRAN.R-project.org/package=maptools.

[ece32038-bib-0006] Bivand, R. , N. Lewin‐Koh , E. Pebesma , E. Archer , A. Baddeley , H.‐J. Bibiko , et al. 2014 maptools: tools for reading and handling spatial objects; CRAN project. Available at: http://r-forge.r-project.org/projects/maptools/.

[ece32038-bib-0007] Blackbridge . 2014 Satellite imagery product specifications. Available at: http://blackbridge.com/rapideye/upload/RE_Product_Specifications_ENG.pdf

[ece32038-bib-0008] Borghesio, L. , and P. Laiolo . 2004 Habitat use and feeding ecology of Kulal White‐eye *Zosterops kulalensis* . Bird Conserv. Int. 14:11–24.

[ece32038-bib-0009] Calenge, C. 2006 The package adehabitat for the R software: a tool for the analysis of space and habitat use by animals. Ecol. Model. 197:516–519.

[ece32038-bib-0010] Carey, A. B. , J. A. Reid , and S. P. Horton . 1990 Spotted owl home range and habitat use in southern Oregon Coast Ranges. J. Wildl. Manage. 54:11–17.

[ece32038-bib-0011] Day, M. D. , C. J. Wiley , J. Playford , and M. P. Zalucki . 2003 Lantana – current management status and future prospects. ACIAR Monograph 102. Canberra

[ece32038-bib-0012] Del Hoyo, J. , A. Elliott , J. Sargatal , D. A. Christie , and de Juana E. (eds.) 2014 Handbook of the birds of the world alive. Lynx Editions, Barcelona, pp. 903.

[ece32038-bib-0013] Dennis, R. L. H. , and H. T. Eales . 1997 Patch occupancy in *Coenonympha tullia* (Muller, 1764) (Lepidoptera: Satyrinae): habitat quality matters as much as patch size and isolation. J. Insect Conserv. 1:167–176.

[ece32038-bib-0014] Denoel, M. , and G. F. Ficetola . 2015 Using kernels and ecological niche modelling to delineate conservation areas in an endangered patch‐breeding phenotype. Ecol. Appl.: 1922–1931. doi:10.1890/14‐1041.1. 26591457 10.1890/14-1041.1

[ece32038-bib-0015] Devictor, V. , R. Julliard , D. Couvet , and F. Jiguet . 2008 Birds are tracking climate warming, but not fast enough. Proc. Biol. Sci. 275:2743–2748. 18713715 10.1098/rspb.2008.0878 PMC2605823

[ece32038-bib-0016] Enanga, E. M. , W. A. Shivoga , C. Maina‐Gichaba , and I. F. Creed . 2011 Observing changes in riparian buffer strip soil properties related to land use activities in the River Njoro Watershed, Kenya. Water Air Soil Pollut. 218:587–601.

[ece32038-bib-0017] Fahrig, L. 2003 Effects of habitat fragmentation on biodiversity. Annu. Rev. Ecol. Evol. Syst. 34:487–515.

[ece32038-bib-0018] Gese, E. M. , D. E. Andersen , and O. J. Rongstad . 1990 Determining home range size of resident coyotes from point and sequential locations. J. Wildl. Manage. 54:501–506.

[ece32038-bib-0019] Ghaffari, H. , F. Ihlow , M. V. Plummer , M. Karami , N. Khorasani , B. Safei‐Mahroo , et al. 2014 Home rage and habitat selection of the endangered Euphrates softshell turtle *Rafetus euphraticus* in a fragmented habitat in southwestern Iran. Chelonian Conserv. Biol. 13:202–215.

[ece32038-bib-0100] Goslee, S. C. 2011 Analyzing Remote Sensing Data in R: The landsat Package. Journal of Statistical Software 43:1–25.22003319

[ece32038-bib-0020] Habel, J. C. , J. Hillen , T. Schmitt , and C. Fioscher . 2016 Restricted movements and high site fidelity in three East African cloud forest birds. J. Trop. Ecol. 32:83–87.

[ece32038-bib-0021] Hansbauer, M. M. , I. Storch , R. G. Pimentel , and J. P. Metzger . 2008 Comparative range use by three Atlantic Forest understorey bird species in relation to forest fragmentation. J. Trop. Ecol. 24:291–299.

[ece32038-bib-0022] Harris, S. , W. J. Cresswell , P. G. Forde , W. J. Trewhella , T. Woollard , and S. Wray . 1990 Home range analysis using radio‐tracking data − a review of problems and techniques particularly as applied to the study of mammals. Man. Rev. 20:97–123.

[ece32038-bib-0023] Hijmans, R. J. 2015 raster: geographic data analysis and modeling. R package version 2.3‐40. Available at: http://CRAN.R-project.org/package=raster.

[ece32038-bib-0025] Hodgson, T. 2013 Radio telemetry tracking, triangulation from two known points using an excel spreadsheet. Available at: http://Blog.Privatei.com.au.

[ece32038-bib-0027] Jackson, R. D. , and A. R. Huete . 1991 Interpreting vegetation indices. Prev. Vet. Med. 11:185–200.

[ece32038-bib-0028] Jacobs, J. 1974 Quantitative measurement of food selection. A modification of the forage ratio and Ivlev's electivity index. Oecologia 14:413–417. 10.1007/BF00384581 28308662

[ece32038-bib-0029] Jeltsch, F. , D. Bonte , G. Péer , B. Reineking , P. Leimgruber , N. Balkenhol , et al. 2013 Integrating movement ecology with biodiversity research – exploring new avenues to address spatiotemporal biodiversity dynamics. Mov. Ecol. 1:6. 25709820 10.1186/2051-3933-1-6 PMC4337763

[ece32038-bib-0030] Kenward, R. 2001 A manual for wildlife radio tagging. Acad. Press, London, San Diego.

[ece32038-bib-0031] Kenya National Bureau of Statistics (1999 and 2009) Kenya Population and Housing Census. Available at: http://www.knbs.or.ke/ (accessed 30 September 2014).

[ece32038-bib-0032] Kernohan, B. J. , R. A. Gitzen , and J. J. Millspaugh . 2001 Analysis of animal space use and movements Pp. 125–166 *in* Millspaugh J. J. and Marzluff J. M., eds. Radio tracking and animal populations. Academic Press, San Diego, CA.

[ece32038-bib-0033] Lindenmayer, D. B. , J. Fischer , A. Felton , M. Crane , D. Michael , C. MacGregor , et al. 2008 Novel ecosystems resulting from landscape transformation create dilemmas for modern conservation practice. Conserv. Lett. 1:129–135.

[ece32038-bib-0034] Lyons, E. E. . 2000 Preliminary survey of invasive species in Eastern Africa. Invasive species in Eastern Africa: Proceedings of a workshop held at ICIPE, July 5–6, 1999 (ed. by E.E. Lyons and S.E. Miller), pp. 65–70. ICIPE Science Press, Nairobi.

[ece32038-bib-0035] McClanahan, T. R. , and T. P. Young . 1996 East African ecosystems and their conservation. Oxford Univ. Press, New York.

[ece32038-bib-0036] Ministry of Agriculture . 2006 Farm management handbook of Kenya Part C, 2nd edn Nairobi Available at: http://star-www.giz.de/fetch/4Q0jm4X0001l0gcgNl/07-1293.pdf.

[ece32038-bib-0037] Moore, R. P. , W. D. Robinson , I. J. Lovette , and T. R. Robinson . 2008 Experimental evidence for extreme dispersal limitation in tropical forest birds. Ecol. Lett. 11:1–9. 18513315 10.1111/j.1461-0248.2008.01196.x

[ece32038-bib-0038] Munger, J. C. 1984 Home ranges of horned lizards (*Phrynosoma*): circumscribed and exclusive? Oecologia 62:351–360. 10.1007/BF00384267 28310888

[ece32038-bib-0039] Newmark, W. D. , V. J. Mkongewa , and A. D. Sobek . 2010 Ranging behaviour and habitat selection of terrestrial insectivorous birds in north‐east Tanzania: implications for corridor design in Eastern Arc Mountains. Anim. Conserv. 13:474–482.

[ece32038-bib-0040] Njoroge, P. , L. A. Bennun , and L. Lens . 1998 Habitat use by the globally endangered Hinde's Babbler *Turdoides hindei* and its sympatric relative, the Nothern Pied Babbler *T. hypoleucus* . Bird Conserv. Int. 8:59–65.

[ece32038-bib-0041] Nychka, D. , R. Furrer , and S. Sain . 2015 Fields: tools for spatial data. R package version 8.2‐1. Available at: http://CRAN.R-project.org/package=fields

[ece32038-bib-0042] Ortiz, S. M. , J. Breidenbach , and G. Kändler . 2013 Early detection of Bark beetle green attack using TerraSAR‐X and RapidEye Data. Remote Sens. 5:1912–1931.

[ece32038-bib-0043] Phillips, S. J. , R. P. Anderson , and R. E. Shapire . 2006 Maximum entropy modeling of species geographic distributions. Ecol. Model. 190:231–259.

[ece32038-bib-0044] Pinheiro, J. C. and D. M. Bates . 2000 Mixed‐effect models in S and S‐plus. Springer‐Verlag New York, New York, USA, 355pp.

[ece32038-bib-0045] Pinheiro, J. , D. Bates , S. DebRoy , D. Sarkar , R Development Core Team . 2012 nlme: Linear and Nonlinear Mixed Effects Models.R package version 3, 1–104, http://CRAN.R-project.org/package=nlme.

[ece32038-bib-0046] QGIS Development Team . 2014 QGIS Geographic Information System. Open Source Geospatial Foundation Project; Available at: http://qgis.osgeo.org.

[ece32038-bib-0444] R Development Core Team . 2013 R: A language and environment for statistical computing. R Foundation for Statistical Computing, Vienna, Austria. ISBN 3‐900051‐07‐0, URL http://www.R-project.org.

[ece32038-bib-0048] Robles, H. , and C. Ciudad . 2012 Influence of habitat quality, population size, patch size, and connectivity on patch‐occupancy dynamics of the Middle Spootted Woodpecker. Conserv. Biol. 26:284–293. 22268847 10.1111/j.1523-1739.2011.01816.x

[ece32038-bib-0049] Sala, O. E. , F. Stuart Chapin , J. J. Armesto , E. Berlow , J. Bloompeld , R. Dirzo , et al. 2000 Global biodiversity scenarios for the Year 2100. Science 287:1770–1774. 10710299 10.1126/science.287.5459.1770

[ece32038-bib-0050] Schneider, C. 2003 The influence of spatial scale on quantifying insect dispersal: an analysis of butterfly data. J. Ecol. Entomol. 28:252–256.

[ece32038-bib-0051] Shaw, P. , J. Musina , and P. Gichuki . 2003 Estimating change in the geographical range and population size of Hinde's Babbler *Turdoides hindei* . Bird Life Int. 13:1–12.

[ece32038-bib-0052] Shaw, P. , P. Njoroge , V. Otieno , and E. Mlamba . 2013 Detecting change in the status and habitat of Hinde's Babbler *Turdoides hindei*, 2000 to 2011. Int. J. Avian Sci. 155:428–429.

[ece32038-bib-0053] Smith, M. J. , M. G. Betts , G. J. Forbes , D. G. Kehler , M. C. Bourgeois , and S. P. Flemming . 2011 Independent effects of connectivity predict homing success by northern flying squirrel in a forest mosaic. Landscape Ecol. 26:709–721.

[ece32038-bib-0054] Spencer, S. R. , G. N. Cameron , and R. W. Swihart . 1990 Operationally defining home range: temporal dependence exhibited by hispid cotton rats. Ecology 71:1817–1822.

[ece32038-bib-0055] Sutherland, W. J. , A. S. Pullin , P. M. Dolman , and T. M. Knight . 2004 The need for evidence‐based conservation. Trends Ecol. Evol. 19:305–308. 16701275 10.1016/j.tree.2004.03.018

[ece32038-bib-0056] Teucher, M. , C. Fischer , C. Busch , M. Horn , J. Igl , J. Kerner , et al. 2015 A Kenyan endemic bird species *Turdoides hindei* at home in invasive thickets. Basic Appl. Ecol. 16:180–188.

[ece32038-bib-0057] Van Der Wal, J. , L. Falconi , S. Januchowski , L. Shoo , and C. Storlie . 2014 SDMTools: species distribution modelling tools: tools for processing data associated with species distribution modelling exercises. R package version 1.1‐221. Available at: http://CRAN.R-project.org/package=SDMTools.

[ece32038-bib-0058] Venables, W. N. , and B. D. Ripley . 2002 Modern applied statistics with S, 4th edn Springer, New York.

[ece32038-bib-0059] Watson, J. E. M. , R. J. Whittaker , and D. Freudenberger . 2005 Bird community responses to habitat fragmentation: how consistent are they across landscapes. J. Biogeogr. 32:1353–1370.

[ece32038-bib-0060] Wiktander, U. , O. Olsson , and S. G. Nilsson . 2001 Seasonal variation in home‐range size, and habitat area requirement of the Lesser Spotted Woodpecker (*Dendrocopos minor*) in southern Sweden. Biol. Conserv. 100:387–395.

[ece32038-bib-0061] Worton, B. J. 1995 Using Monte Carlo simulation to evaluate kernel‐based home range estimators. J. Wildl. Manage. 59:794–800.

[ece32038-bib-0062] Zar, J. H. . 1996 Biostatistical analysis, 3rd edn Prentice Hall International, London.

[ece32038-bib-0063] Zimmerman, D. A. , D. A. Turner , and D. J. Pearson . 1996 Birds of Kenya and northern Tanzania. Princeton Univ. Press, Princeton, NJ.

