# Supplementary material for: Kenyan endemic bird species at home in novel ecosystem
Source: Ecol Evol. 2016 Mar 14;6(8):2494–505. doi: 10.1002/ece3.2038 (PMC4797158; doi:10.1002/ece3.2038)

**Appendix** Fig. A1. Home range area (in ha) per individual against sample size (days after start of data acquisition), shown for a) Minimum Convex Polygon estimator using 95% of the relocations (MCP95) in August 2014; b) Kernel home ranges for 95% levels (K95) in August 2014, c) MCP95 in February/ March 2015, and d) K95 in February/ March 2015. The different species are abbreviated with ZG for *A. importunus insularis,* BT for *T. tephronotus,* RC for *T. rubiginosus,* and HB for *T. hindei.*


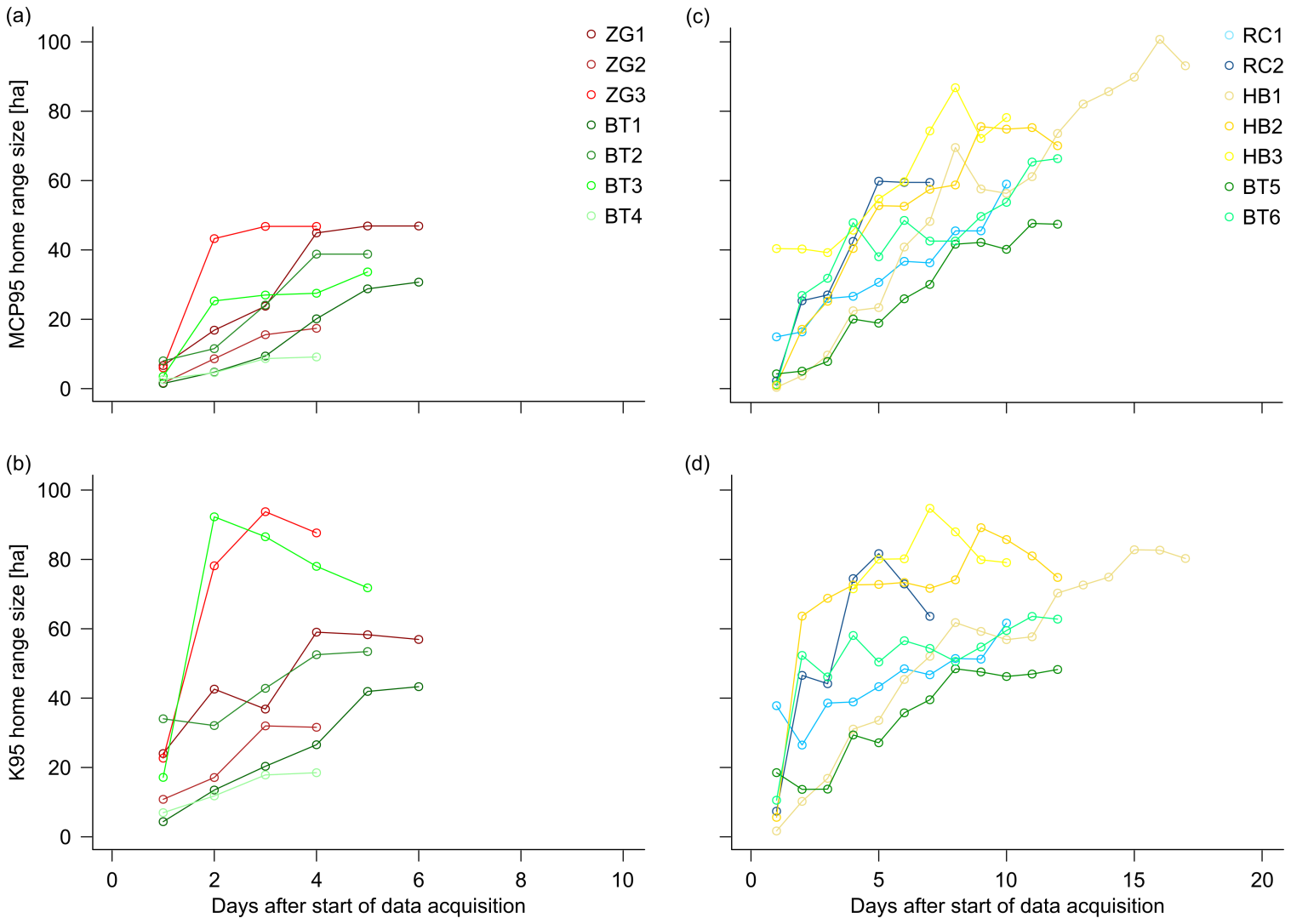

Supplement: Supplementary file 1 — Figure S1. Home range area (in ha) per individual against sample size (days after start of data acquisition), shown for (a) Minimum Convex Polygon estimator using 95% of the relocations (MCP95) in August 2014; (b) Kernel home ranges for 95% levels (K95) in August 2014, (c) MCP95 in February/ March 2015, and (d) K95 in February/ March 2015. [file ECE3-6-2494-s001.docx]
